# Single Co_3_O_4_ Nanocubes Electrocatalyzing the Oxygen Evolution Reaction: Nano-Impact Insights into Intrinsic Activity and Support Effects

**DOI:** 10.3390/ijms222313137

**Published:** 2021-12-04

**Authors:** Zhibin Liu, Manuel Corva, Hatem M. A. Amin, Niclas Blanc, Julia Linnemann, Kristina Tschulik

**Affiliations:** Analytical Chemistry II, Faculty of Chemistry and Biochemistry, Ruhr University Bochum, 44801 Bochum, Germany; zhibin.liu@rub.de (Z.L.); manuel.corva@rub.de (M.C.); hatem.abdelhalim@rub.de (H.M.A.A.); niclas.blanc@rub.de (N.B.); julia.linnemann@rub.de (J.L.)

**Keywords:** single-entity electrochemistry, Co_3_O_4_, oxygen evolution reaction, nanoparticle, support effect

## Abstract

Single-entity electrochemistry allows for assessing electrocatalytic activities of individual material entities such as nanoparticles (NPs). Thus, it becomes possible to consider intrinsic electrochemical properties of nanocatalysts when researching how activity relates to physical and structural material properties. Conversely, conventional electrochemical techniques provide a normalized sum current referring to a huge ensemble of NPs constituting, along with additives (e.g., binders), a complete catalyst-coated electrode. Accordingly, recording electrocatalytic responses of single NPs avoids interferences of ensemble effects and reduces the complexity of electrocatalytic processes, thus enabling detailed description and modelling. Herein, we present insights into the oxygen evolution catalysis at individual cubic Co_3_O_4_ NPs impacting microelectrodes of different support materials. Simulating diffusion at supported nanocubes, measured step current signals can be analyzed, providing edge lengths, corresponding size distributions, and interference-free turnover frequencies. The provided nano-impact investigation of (electro-)catalyst-support effects contradicts assumptions on a low number of highly active sites.

## 1. Introduction

The sluggish kinetics of the oxygen evolution reaction (OER) are decisive for reaction rates and energy efficiency in sustainable technologies such as water-splitting electrolyzers and metal–air batteries [1]. Hence, designing highly electrocatalytically active, low-cost nanomaterials for the OER has become a central aspect of energy research [2]. Systematic and rapid progress relies on a profound understanding of factors determining catalytic activity, enabling the development of suitable non-noble catalysts such as cobalt oxide nanomaterials. This concerns two major aspects: (1) reliably correlating (structural) material properties to electrocatalytic activities and (2) taking essential components of an electrolyte/electrode system into consideration, which may be interrelated with electrocatalytic activity. Recently, the intrinsic activity of single Co_3_O_4_ nanoplatelets was investigated using scanning electrochemical cell microscopy (SECCM) as well as the single-particle-at-the-tip approach [3,4,5]. The local resolution of SECCM was also used to probe the catalytic response of surface-immobilized β-Co(OH)_2_ nanoplatelets one-by-one [6]. However, analysis of nanocubes or nanospheres has not yet been achieved this way.

Herein, we establish the approach of nano-impact electrochemistry for cube-shaped nanoparticles (NPs), assessing statistical information on size and related intrinsic catalytic activity of single particles. The turnover frequencies (TOFs) determined for individual nanocubes at several potentials are in the range of 10^4^ s^−1^, which is an order-of-magnitude higher than for conventional rotating disc electrode (RDE) measurements. Such characterization approaches employing NP-coated electrodes suffer from ensemble effects due to interparticle interactions, which often conceal the intrinsic nanocatalyst activity [7,8].

The nano-impact technique studies dispersed NPs in electrolyte solution, which collide with a potentiostated microelectrode and alter the recorded current signal [8,9,10]. To enable the investigation of cubic particles, we employ finite element simulations and derive a relationship between measured steady-state current and edge length of a cube.

Due to the well-defined system formed of single NPs electrocatalyzing oxygen evolution, reliable characterization data and respective conclusions on, e.g., structure–activity relationships can be extracted. We further introduce a strategy to probe support effects in electrocatalysis by nano-impacts, allowing us to gain mechanistic insights. Different support (current collector) materials were tested by using two different target electrodes at which Co_3_O_4_ nanocubes collide. In contrast to the high current densities and TOFs achieved on Pt microelectrodes, carbon fiber impact currents were much smaller. The corresponding investigation excludes support effects that only very locally affect catalytic OER activity, e.g., hydrogen transfer to support-related sites. Simulating the diffusional flux towards single NPs clearly shows that a small region of high activity close to the support could not account for the measured currents.

## 2. Results and Discussion

**Figure 1:**
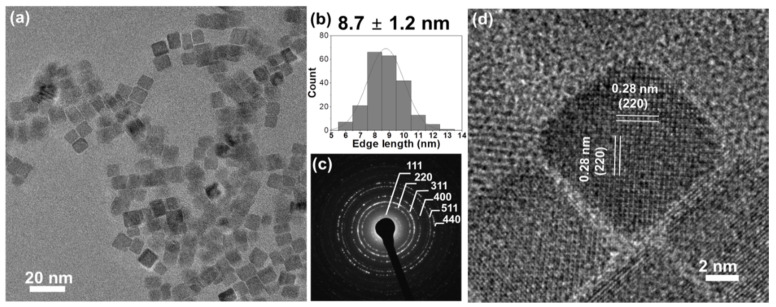
(**a**) Overview TEM and (**d**) high resolution TEM images; (**b**) edge-length histogram (including the mean value of edge length ± standard deviation) and (**c**) SAED pattern of Co_3_O_4_ cubes.

Co_3_O_4_ nanocubes were synthesized by a hydrothermal method, heating a 16 mM cobalt (II) acetate solution at 150 °C for 1 h. The produced cobalt oxide forms uniformly sized cubes with an edge length of 8.7 ± 1.2 nm, as characterized by transmission electron microscopy (TEM, Figure 1a,b). The selected area electron diffraction (SAED) pattern (Figure 1c) indicates phase-pure spinel Co_3_O_4_ (JCPDS no. 42-1467) [11]. High-resolution TEM images (Figure 1d) show a lattice fringe spacing of 0.28 nm, corresponding to (220) planes. Hence, the dominant exposed facets are {100}, as expected for cubes [12].

**Figure 2:**
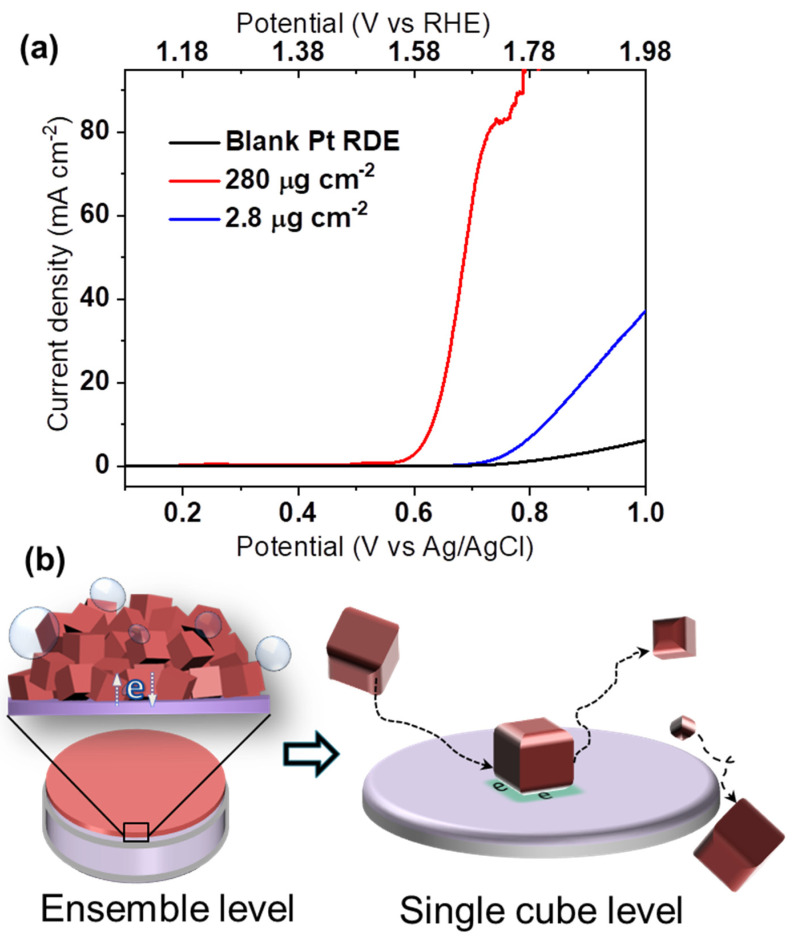
(**a**) LSV curves for 280 μg cm^−2^ and 2.8 μg cm^−2^ of Co_3_O_4_ cubes loaded on a Pt RDE without further additives at 1600 rpm in aqueous 0.1 M KOH, (**b**) schematic representation of electrocatalytic ensemble and nano-impact characterization.

The remarkable OER activity of the yielded monodispersed spinel Co_3_O_4_ nanocubes was initially characterized by a conventional ensemble study (Figure 2a). Using a Pt RDE in aqueous 0.1 M KOH solution, the effect of the catalyst mass loading was examined. Although higher loading nominally increases the total number of active sites, the associated activity increase may be strongly impaired by, e.g., poor electric contact within a thicker catalyst film. In Figure 2a, the mass loading is increased 100 fold, while the measured catalytic current fails to increase proportionally. This exemplifies that normalized electrocatalyst activities are usually inferred from ensemble effects, which must be holistically addressed and understood [13]. Inaccurate structure–activity relationships may easily be concluded from such experiments, and comparison between different studies can be challenging [14,15].

As recently reported, the material that supports NPs can greatly affect their electrocatalytic response [16,17,18,19,20]. Investigating this influence, we find nanocube-coated Pt RDEs more catalytically active than glassy carbon-based ones (Figure S4). However, examining the indicated support effect and measuring at application-relevant current densities is not feasible, e.g., due to elevated gas bubble evolution (Figure 2a). Thus, platinum and carbon fibre microdisc electrodes were loaded with Co_3_O_4_ cubes. The defined and enhanced convergent diffusion characteristics of microelectrodes enable kinetic characterization, while avoiding mechanical stress and ohmic drop issues [21]. The high electrocatalytic activity of the nanocubes at Pt allows to then reach the mass-transport-limited linear sweep voltammetry (LSV) region above ca. 1.0 V vs. Ag/AgCl (=1.98 V vs. reversible hydrogen electrode, RHE) whereas an increasing current is still observed at 1.15 V for the carbon fibre microdisc support (Figure S5). Again, an effect of the support material is suggested but difficult to elucidate further using the ensemble approach. Moreover, the mass loading of NP ensembles on the microelectrodes could not be controlled sufficiently. Accordingly, the intrinsic electrocatalytic activities of the Co_3_O_4_ nanocubes in regard to support effects are investigated by nano-impact electrochemistry.

As depicted in Figure 2b, the nano-impact method makes it possible to overcome limitations of ensemble studies by assessing intrinsic OER activity at the single NP level during “nano impacts” at a microelectrode. In brief, electron transfer (and so, OER) occurs only when a colloidal Co_3_O_4_ nanocube catalyst reaches the surface of a potentiostated target microelectrode, e.g., via Brownian motion. The resulting current transient relates to OER-electrocatalysis at the NP, and accordingly, exhibits a step-like shape for a temporary stay of the NP at the electrode [22].

**Figure 3:**
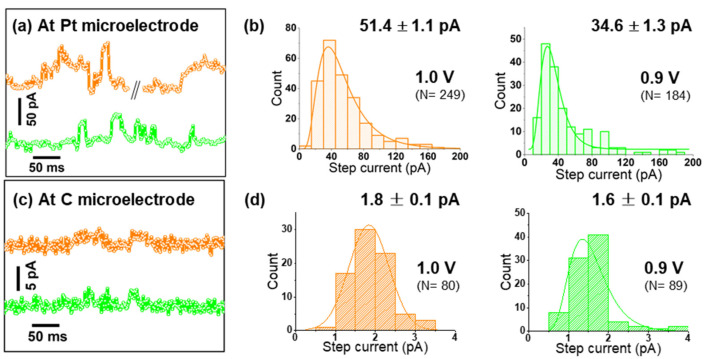
(**a**,**c**) Representative chronoamperograms (offset for better comparison) pertaining a Pt or carbon microelectrode, respectively; (**b**,**d**) step-current histograms of Co_3_O_4_ nanocubes impacting a Pt or carbon microelectrode at 0.9 and 1.0 V, see Figure 4c,d for data obtained at additional potentials.

**Figure 4:**
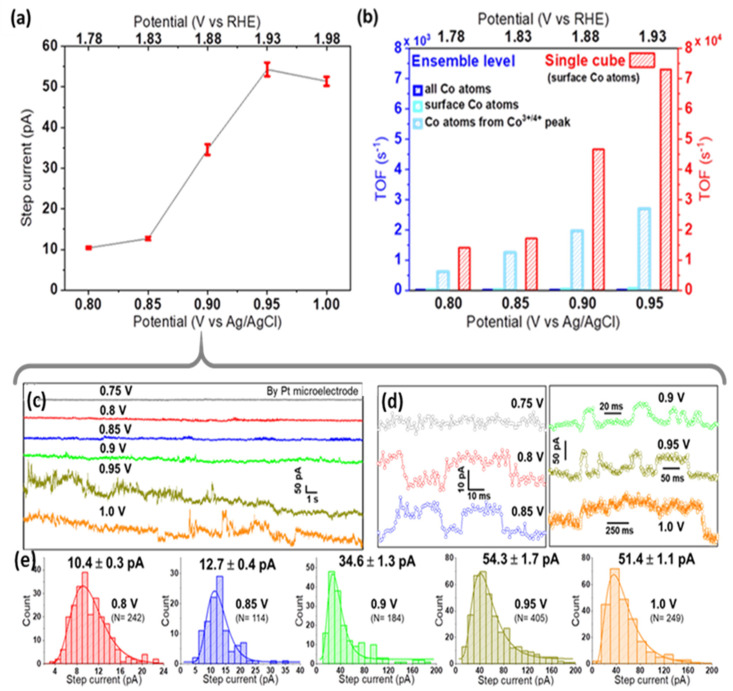
(**a**) Current–potential response in nano-impact experiments, showing the mean ± std. error of the mean step current, (**b**) TOF comparison for Co_3_O_4_ cubes probed at ensemble vs. single cube level. For calculations at the ensemble level, either all Co atoms, surface Co atoms, or Co atoms contributing to the Co^3+/4+^ oxidation peak of the LSV (peak prior to OER ‘onset’) were considered as active sites of 2.8 μg cm^−2^ nanocubes drop-cast on a Pt RDE. Note the 10-times larger scale for single cubes; representative chronoamperograms (**c**) and enlarged impact signals (**d**) recorded at different potentials (offset vertically for better comparison), and (**e**) the obtained step-current histograms used for plot (**a**).

As seen in Figure S6, Pt microelectrodes are comparably inactive towards OER in alkaline solutions. Moreover, on Pt, a peak-to-peak noise level of ca. 7 pA was observed at low applied potentials, while at the highest applied bias, it reached up to 15 pA (Figure S7). This noise level is sufficiently smaller than the nano-impact signals at each specific potential, and thus confirms the suitability of Pt microelectrodes for nano-impact experiments [23]. Such impact measurements were carried out by immersing a Pt microelectrode in a dispersion of Co_3_O_4_ cubes in aqueous 0.1 M KOH and applying constant potentials in the range from 0.75 to 1.0 V vs. Ag/AgCl. Representative examples of the measured current transients are given in Figure 3a and Figure 4c,d. Step-like peaks are observed in the potential range of 0.8–1.0 V (Figure 4d), while current traces recorded at 0.75 V exhibit an insufficient signal-to-noise ratio for detecting clearly recognizable steps (Figure S8). Step-current histograms (Figure 3b and Figure 4e) at various potentials were built from a large number of detected steps (N) and fitted to obtain the corresponding mean values for step currents as a function of applied potential. In Figure 4a, it can be clearly seen that the step current increases slightly from 0.8 to 0.85 V, while a much higher value is obtained at 0.9 V. Due to this strong potential dependence, we conclude that on Pt microelectrodes, kinetics limit the current at potentials ≤0.85 V. Approaching 0.95 V, the Co_3_O_4_ cube current reaches a limiting steady-state value, as indicated by the plateau above 0.95 V in Figure 4a.

Previous investigations on spherical CoFe_2_O_4_ NPs suggest that the nano-impact steady-state current is limited by oxygen diffusion instead of OH^−^ transport. This steady-state current can be calculated according to well-established equations if the correct reactant bulk concentration and diffusion coefficient are considered [24]. The diffusion limited currents towards cubic NPs on a surface were estimated from random walk simulations [25] and for free cubic particles from finite difference simulations [26]. To our knowledge, however, there is not yet an equation describing diffusional steady-state currents for impacting cubic particles at hand. Finite element simulations performed to provide this equation (see Supplementary Information (SI), section SI-I) yielded:

(1) $$I_{\mathrm{ss}}=5.4e_{c}c_{O_{2}}D_{O_{2}}zF$$

where $I_{\mathrm{ss}}$ is the steady-state current, $e_{c}$ the edge length of a cube, $c_{O_{2}}$ the concentration difference between electrode and bulk of the diffusing species (oxygen), $D_{O_{2}}$ is its diffusion coefficient, z the number of transferred electrons and F the Faraday constant.

Based on this equation, the step-current histograms obtained at potentials establishing steady-state diffusion can be converted to “electrochemically measured” size distributions (Figure S9a). At 0.95 V, the steady-state condition is reached, while background oxygen production is still minimal (see SI, section SI-II). The mean value of the calculated edge length is 12.5 nm (at 0.95 V, Figure S9a), exceeding TEM estimations (8.7 nm). However, while TEM-derived sizes result from the investigation of immobilized nanoparticles, nano-impact sizing is affected by the complexity of dynamic systems in solution, as discussed in section SI-II of the SI. Notably, disc centrifuge measurements of colloidal cubes suspended in 0.1 M KOH suggest an average size of 13 to 16 nm (Figure S9b). This may result from its sensitivity to the hydrodynamic radius, rather than to the oxide core as for TEM. Remarkably, ex situ TEM imaging performed after OER (Figure S10) indicates unaltered shape and size of the nanocubes upon OER catalysis. Therefore, the results support that no significant agglomeration occurs in the electrolyte and under OER conditions, validating the quality of the nano-impact dataset. Within the observed typical experimental deviations, good agreement between the average NP sizes is achieved, experimentally supporting the numerically derived equation for diffusional mass transport at impacting nanocubes.

To assess the intrinsic activity of Co_3_O_4_ cubes, the activity metric TOF (electrons transferred per active site per second at a given potential) is calculated (see details in SI, section SI-III). We consider all electrolyte-exposed surface Co atoms of a nanocube as potential active sites, since the current in nano-impact experiments originates from the full electrolyte-exposed cube surface thanks to the avoidance of binders, etc. Calculations are based on TEM-derived size information and various approaches to estimate the number of active sites for ensembles [27] of different mass loadings. The intrinsic activity obtained for single Co_3_O_4_ nanocubes in impact studies (TOF = 7.3 × 10^4^ s^−1^ at 0.95 V) exceeds measures derived from ensemble data by at least one order of magnitude (Figure 4b). Ensemble effects discussed earlier obviously affect determined TOF values, indicating shortcomings of such conventional characterization measurements when comparing electrocatalytic activities of different nanomaterials.

Support effects at the single-entity level were then investigated by varying the material of the target microelectrode, which represents the supporting material providing electrical contact to an individual impacting nanocatalyst. Performing identical experiments as before with a commercial carbon microelectrode, nano-impact signals were detected only at relatively high potentials (0.9 and 1.0 V vs. Ag/AgCl, Figure 3b and Figure S11) and with negligible intensities. This detection was possible only due to the much smaller noise level observed on carbon (ca. 2 pA peak-to-peak). Comparing Figure 3a–d, an almost 30-times larger average impact current is revealed for Pt microelectrodes at 1.0 V.

Support effects on electrocatalytic activity have previously been researched for film and NP ensemble electrodes, deriving several hypotheses about underlying mechanisms [28,29,30]. Substantial progress on the subject requires us to identify whether support materials indirectly affect the reaction kinetics of the OER, for instance by enhancing the transformation of a precatalyst to a more active phase, or if catalyst–support interactions have a direct effect, e.g., by providing additional, synergistic adsorption sites for reactants. Direct support effects on OER kinetics would lead to locally very high catalytic currents directly where the NP interfaces the support material and the electrolyte. E.g., Frydendal [29] et al. ascribed the direct enhancement effect of gold near Co_3_O_4_ catalysts to Au=O sites accepting hydrogen, and thus stabilizing *OOH intermediates, at a small subset of Co sites. Regarding identified Pt^δ+^ sites between Pt and Co_3_O_4_ for atomically defined catalysts, [31] interfacial electron transfer to metal centres in the oxide might also cause similarly improved OER-behaviour as that discussed for metal–support interactions at iridium oxide [30].

**Figure 5:**
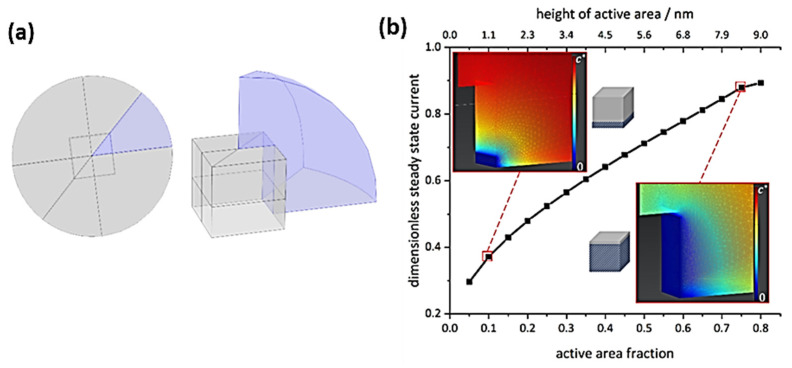
(**a**) Schematic representation of the model geometry (not true to scale) used for simulating the diffusional flux towards a single nanocube. Here, the magnitude of the steady state flux is equal for diffusion towards and away from an electrode, hence only diffusion profiles towards the electrode are shown. (**b**) Respective dimensionless steady-state currents for varying active areas, normalized to Equation (1) with five active cube faces and steady-state concentration gradients, including illustrations for varied partial cube activity, see SI section SI-III for details.

To evaluate such assumptions about direct local support effects on OER kinetics, the diffusional flux at an impacting individual nanocube was quantitatively assessed by simulations (Figure 5). Details on simulations are presented in the SI, section SI-I. As exemplified by the concentration profiles, a considerable amount of the cube surface must be highly activated to cause fluxes which account for the experimentally observed currents. Even if local support effects could accelerate OER kinetics to reach maximum concentration gradients at 10% of the cube surface, this would only correspond to <40% of the current expected for the whole cube surface being highly active. Thus, we conclude that direct support effects, very locally affecting catalytic OER activity, are not the cause of the activity increase observed in our study. The reason why the Co_3_O_4_ nanocubes supported on Pt show such an increased response with respect to the carbon-supported ones must relate to a different phenomenon, involving on average the whole exposed cube surface.

An indirect effect of a support may rely on the reversible transformation of a (sub-) nanometer-thick layer of the oxide catalyst under OER conditions [13]. Such amorphized layers were identified as the active catalytic phase of Co_3_O_4_ at thin film and ensemble electrodes [32,33]. In line with these ensemble results, our single-entity study suggests that the Pt support enhances the conversion of the cube surface layer to the active phase, and thus increases the activity across the full catalyst surface instead of just near-support Co sites, since our simulation revealed that the latter would result in a significant decrease in overall current per impact signal. Further, scanning tunnelling microscopy and X-ray photoelectron spectroscopy investigations of model catalysts showed that a supporting gold substrate shifts the transformation of cobalt oxide surfaces to an OER-active cobalt-oxyhydroxide phase to lower overpotentials [30].

Hence, the deviating electrocatalytic currents at platinum and carbon target electrodes may correspond to electrochemical conversion of the cobalt oxide cube surface. An impacting Co_3_O_4_ NP is then understood as a precatalyst whose surface activation is triggered at the metallic support/catalyst/electrolyte boundary region and propagates along the catalyst/electrolyte interface. Previously mentioned effects of electron density distribution or additional acceptor sites might, then, facilitate the initial surface transformation instead of directly affecting OER kinetics [20]. Note that carbon corrosion can take place under oxidative conditions, possibly resulting in surface changes such as roughening or exfoliation of graphene-like layers [34]. However, respective investigations (Figure S12) indicated that the observed difference between carbon and Pt target microelectrodes is not due to carbon corrosion. Figure S12b shows recognizable oxidative current at carbon electrodes for chronoamperometry at 1.2 V but not at 1.0 V.

## 3. Materials and Methods

### 3.1. Chemicals

Cobalt acetate tetrahydrate (Alfa Aesar, Kandel, Germany, metal basis 99.999%), triethylene glycol (TEG, Sigma-Aldrich, Darmstadt, Germany, 99%), potassium hydroxide (Alfa Aesar, Kandel, Germany, 85% min., metal basis 99.99%) and 1,1′-ferrocenedimethanol (Acros Organics, Nidderau, Germany, 98%) were used as received.

### 3.2. Preparation of Co_3_O_4_ Nanocubes

Cobalt acetate tetrahydrate (0.1 g) was dissolved in a mixture of 20 mL H_2_O and 5 mL TEG transferred into a 150 mL glass autoclave. After sealing, it was heated in a preheated 150 °C oil bath under continuous stirring for 1 h. Afterwards, the obtained brown slurry was centrifuged at 11,000 rpm (Eppendorf) for 10 min and resuspended in H_2_O. This work-up procedure was repeated twice, removing the unreacted salt and TEG. Finally, the precipitate was collected after drying overnight at 60 °C.

### 3.3. Materials Characterization

Transmission electron microscopy (TEM) and selected area electron diffraction (SAED) were carried out on a JEOL JEM-2200FS field emission electron microscope operating at 200 kV. Samples for TEM studies were prepared by drop-casting an aqueous suspension of Co_3_O_4_ cubes onto a carbon coated 200-mesh copper grid. For determining size distributions, a Co_3_O_4_ cube suspension was prepared by dispersing cubes in aqueous 0.1 M KOH solution at the same concentration as used in nano-impact experiments, sonicating for 10 min. After an additional period of 5 min, the Co_3_O_4_ particle size was also determined using a CPS Disc Centrifuge instrument (DC24000 UHR, CPS Instruments Europe, Oosterhout, The Netherlands). Prior to the measurement, a density gradient comprising 24 wt.% and 8 wt.% sucrose solutions was built within the disc, and a disc rotation of 12,000 rpm was used. A calibration standard of poly(vinyl chloride) with a diameter of 483 nm was used. The CPS instrument software was used to obtain the weight-based particle size distribution considering the density of Co_3_O_4_ (6.11 g cm^−^^3^) [35].

### 3.4. Electrochemical Measurements

All electrochemical measurements were performed with a three-electrode system connected to a Bio-Logic SP-300 electrochemical workstation. A Pt foil or a Pt wire were used as counter electrodes and a Ag/AgCl (3 M KCl) electrode acted as the reference electrode. For aqueous 0.1 M KOH electrolyte solution, the measured potentials can be converted into potentials versus reversible hydrogen electrode (RHE) via the equation:

E(RHE) = E(Ag/AgCl) + 0.210 V + 0.059 V × pH = E(Ag/AgCl) + 0.977 V

#### 3.4.1. Ensemble Characterization with Rotating Disc Electrodes (RDEs)

For RDE ensemble measurements, either a glassy carbon (GC) or a Pt disc electrode (ALS) of 3 mm in diameter was used as the working electrode. Before measuring, the working electrode was polished with 1, 0.3 and 0.05 μm Al_2_O_3_ slurries on Buehler pads consecutively and then sonicated in water. Then, 5 µL of a Co_3_O_4_ cube suspension (nanocubes in water with no binder, 4 and 0.04 mg mL^−1^) was loaded onto the working electrode by drop-casting to obtain loadings of 280 μg cm^−2^ and 2.8 µg cm^−2^, respectively. During testing in aqueous 0.1 M KOH solution, the working electrode was continuously rotated at 1600 rpm (ALS, RRDE-3A). Linear sweep voltammograms (LSVs) were recorded at 5 mV s^−1^ with iR compensation (95%, hardware compensation mode in EC-Lab^®^ 11.12, resistance value was determined by impedance technique at 100 kHz and open circuit potential).

#### 3.4.2. Microelectrode Preparation and Characterization

In this study, microelectrodes of Pt (25 µm diameter, in-house made) and carbon fiber (7 µm diameter, ALS) were employed. For the fabrication of a Pt microelectrode, a Pt wire (length ca. 1 cm, 25 µm diameter) was put into a glass capillary and sealed under vacuum. The Pt wire was soldered inside the capillary to a silver wire serving as an electrical contact. The tip of the Pt microelectrode was then polished to obtain a clean and smooth surface.

To test the suitability of such self-made Pt microelectrodes for nano-impact experiments, a Pt microelectrode was used as working electrode to run a blank LSV experiment in aqueous 0.1 M KOH electrolyte at 10 mV s^−1^. After repeating the cleaning procedure, the Pt microelectrode was dipped into 0.04 mg mL^−1^ Co_3_O_4_ cube suspension, dried by a dry stream of nitrogen, and again characterized by LSV. After each measurement, Co_3_O_4_ cubes were removed by polishing the Pt microelectrode on polishing paper and another blank LSV test was run. Furthermore, chronoamperometric blank tests were performed with a freshly cleaned Pt microelectrode at various potentials in the range from 0.75 to 1.0 V vs. Ag/AgCl.

To assess the stability of the Pt and carbon fiber microelectrodes used in this study, chronoamperometric measurements in aqueous 0.1 M KOH solution and cyclic voltammetry (CV) characterization in an aqueous 0.1 M KCl solution with 1 mM ferrocenedimethanol were performed. The chronoamperometric response was recorded at 1.0 and 1.2 V for 5 min. CVs were measured at 25 mV s^−1^ in a potential range from 0 to 0.45 V vs. Ag/AgCl before and after chronoamperometry.

#### 3.4.3. Ensemble Characterization with Microelectrodes

To load Co_3_O_4_ cubes on the Pt and carbon fiber microelectrodes for electrochemical ensemble measurements, the microelectrodes were immersed into 0.04 mg mL^−1^ Co_3_O_4_ cube suspension for ca. 1 min and dried by a dry nitrogen stream. Prior to this coating procedure, the microelectrodes were polished on polishing paper and rinsed with water to provide a clean surface. The electrocatalytic OER activity of the cube ensembles on microelectrodes was characterized by CV at 50 mV s^−1^ in an aqueous solution of 0.6 mM KOH and 0.1 M KCl in a potential range from 0 to 1.15 V vs. Ag/AgCl. Additionally, respective blank CVs were recorded prior to the nanocube loading procedure.

#### 3.4.4. Nano-Impact Experiments Probing Individual Co_3_O_4_ Nanocubes

For nano-impact experiments, the Pt and carbon fiber microelectrodes were used as target working electrodes. The microelectrodes were firstly polished on sandpaper (from 400 to 7000 grit sandpaper) and finally on polishing cloth using a 0.3 μm Al_2_O_3_ slurry. After thoroughly rinsing with water, they were dried under nitrogen flow. All nano-impact measurements were carried out in a double-Faraday-cage system to minimize electronic noise. A Ag/AgCl (3 M KCl) reference electrode and Pt wire counter electrode were inserted into a double junction filled with 0.1 M KOH. This junction was immersed into the electrolyte. Then, 1 µL of aqueous 0.04 mg mL^−1^ Co_3_O_4_ cube suspension was injected into 1 mL of aqueous 0.1 M KOH solution, sonicated for 1 min, and then used to run nano-impact experiments. The concentration of cube particles was 0.016 pM. Chronoamperograms were recorded for 5 min at a data acquisition rate of 1 ms, utilizing the built-in 50 kHz low-pass filter of the Bio-Logic potentiostat.

### 3.5. Finite Element Simulations for Diffusion-Controlled Currents at Cubic Nanoparticles

The steady-state current equation for cubic nanoparticles was determined using the commercial finite element solver COMSOL Multiphysics. Details on simulations are presented in the Supplementary Materials.

## 4. Conclusions

In conclusion, we successfully quantified the intrinsic OER activity of single Co_3_O_4_ cubes in terms of TOF without the concealing interference of conventional ensemble techniques. The nano-impact method significantly enhances the validity of findings. This may enable conclusive comparative studies (catalyst benchmarking) because influencing factors such as material loading are avoided. By investigating the effect of support materials on electrocatalytic activity, we demonstrate that nano-impact measurements can provide insights for nanocatalyst and electrode design. The comparison of NP collision events at Pt and carbon target electrodes suggests that the Pt support promotes the transformation of the Co_3_O_4_ NP surface into a highly active OER-catalysing phase. The provided extension of the nano-impact approach from spherical to cubic NPs may enable new opportunities to investigate structure–activity relations, e.g., by employing nanocubes exposing different crystal facets.

## Data Availability

All experimental supporting data and procedures are available within this article and the Supplementary Materials.

## Supplementary Materials

The following are available online at https://www.mdpi.com/article/10.3390/ijms222313137/s1.
