# Supplementary material for: Single Co3O4 Nanocubes Electrocatalyzing the Oxygen Evolution Reaction: Nano-Impact Insights into Intrinsic Activity and Support Effects
Source: Int J Mol Sci. 2021 Dec 4;22(23):13137. doi: 10.3390/ijms222313137 (PMC8658644; doi:10.3390/ijms222313137)
Supplement: Supplementary file 1 [file ijms-22-13137-s001.zip › ijms-1472725-supplementary.pdf]

Supplementary Information (SI)

Single Co_3O_4 Nanocubes Electrocatalyzing the Oxygen Evolution Reaction: Nano-impact Insights into Intrinsic Activity and Support Effects

Zhibin Liu [1,2], Manuel Corva [1], Hatem M. A. Amin [1], Niclas Blanc [1], Julia Linnemann [1] and Kristina Tschulik [1,*]

[1] Analytical Chemistry II, Faculty of Chemistry and Biochemistry, Ruhr University Bochum, Bochum 44780, Germany; zhibin.liu@rub.de (Z.L.); manuel.corva@rub.de (M.C.); hatem.abdelhalim@rub.de (H.M.A.A.); niclas.blanc@rub.de; julia.linnemann@rub.de (J.L.)

[2] Institute of Zhejiang University-Quzhou, Quzhou, 324003, China

* Correspondence: kristina.tschulik@ruhr-uni-bochum.de

Table of contents

SI-I) Finite element simulations for diffusion-controlled currents at cubic nanoparticles 3

SI-II) Conversion of nano-impact data to size distribution 6

SI-III) Calculation of turnover frequencies (TOFs) 8

SI-III.1. TOFs determined from ensemble studies 8

SI-III.1.1 All Co atoms are accessible for OER 8

SI-III.1.2 Surface Co atoms are accessible for OER 8

SI-III.1.3 Co atoms contributing to the oxidation peak are accessible for OER 9

SI-III.1.4 Summarized TOF values estimated from ensemble studies with a Pt RDE 10

SI-III.2. TOFs determined from nano-impact studies 10

SI-IV) Additional supporting figures 11

Figure S1 11

Figure S2. 12

Figure S3. 13

Figure S4. 14

Figure S5. 15

Figure S6.. 16

Figure S7.. 17

Figure S8. 18

Figure S9. 19

Figure S10.. 20

References 21

SI-I) Finite element simulations for diffusion-controlled currents at cubic nanoparticles

The steady state current equation for cubic nanoparticles was determined using the commercial finite element solver COMSOL Multiphysics. The steady state for a diffusion-controlled flux at a cubic nanoelectrode, which is supported on an inert surface, was simulated. To minimize the number of required mesh elements, one eighth of the geometry was modelled, taking advantage of the available mirror planes, as it is shown in Figure S1.

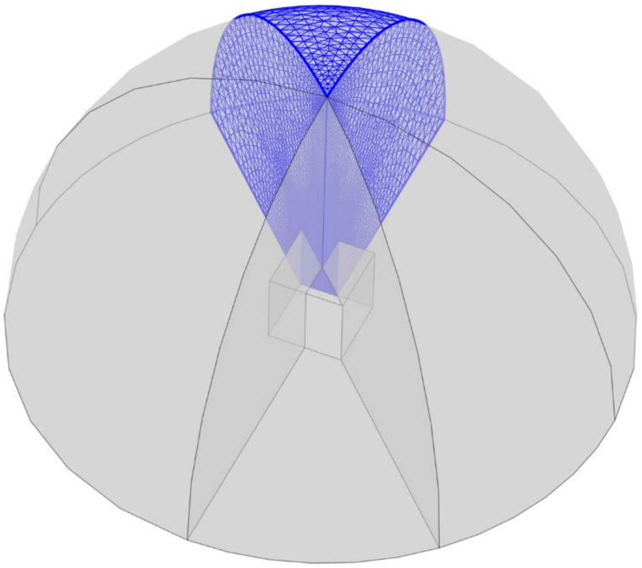

Figure S1. Schematic of the mirror planes used to minimize the cubic geometry and simulate one eights of the total geometry.

All simulations were performed using diffusion only mass transport mechanisms and a stationary solver. The concentration boundary conditions were set to $c = c^*$ at the outer boundary of the geometry and to $c = 0$ at the electrode. The domain equations

$$\frac{\partial c}{\partial t} = -\nabla \boldsymbol{J} \text{ and}$$

$$\boldsymbol{J} = -D\nabla c$$

where $\boldsymbol{J}$ is the diffusional flux and t is time, were used to describe the mass transport between inner and outer domain boundaries. To keep the total number of mesh nodes and general aspect ratio within the model as low as possible, an infinite element domain surrounding an inner domain of 40 times the NP dimension was used. As the expected gradients in this region are fairly low, this node can be used to upscale the distance between the boundaries and increase the total simulated volume. An appropriate mesh density at the electrode was determined by simulations of steady state currents with a hemispherical electrode. In this case, the mesh density was successively increased, until the analytical value for a steady state flux (j_{ss}) at the electrode was obtained, which is given by

$$j_{\text{ss}} = 2\pi rcD$$

where r is the radius of the hemisphere, c and D are the bulk concentration and diffusion coefficient of the consumed species, respectively. For enhanced comparability, a dimensionless flux J can be defined as:

$$J = \frac{j}{j_{\text{ss}}}$$

where j is obtained by integration of the total diffusive flux over the electrode surface. Figure S2 shows the steady-state concentration gradients for the cubic model and the specific hemispherical model, which showed a dimensionless flux of 1.0001.

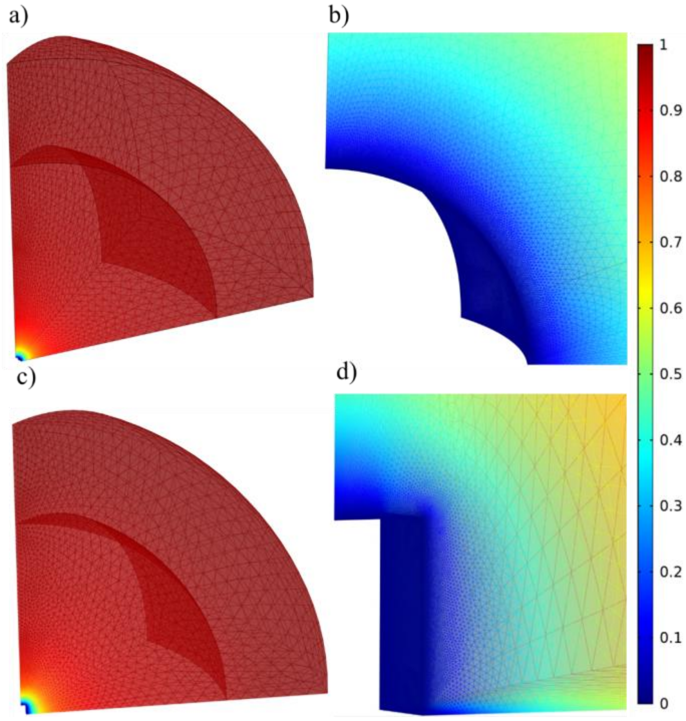

Figure S2. Stationary dimensionless concentrations with a custom mesh for hemispherical (a and b) and cubic (c and d) electrodes. A dimensionless concentration of 1 is the bulk concentration.

Settings for the free tetrahedral mesh, which was used in three dimensional domains are presented in Table S1.

Table S1. Settings for both domains for hemispheric and cubic nanoparticles.

Mesh parameter	Input value
maximum element size	$\frac{10}{3}e_c$
minimum element size	$\frac{e_c}{2000}$
maximum element growth rate	1.1
curvature factor	0.07
resolution of narrow regions	1

The radius of the hemisphere was set to half of the cubic edge length (e_c).

Special mesh settings were used for the surface characterizing the electrode and along electrode edges, where a set number of elements were defined. At the cubic electrode, 800 and 400 mesh elements were set for vertical and horizontal edges respectively. The rest of the modified settings for the electrode are presented in Table S2.

Table S2. Settings for the electrode surface boundaries, which were used in simulations for hemispherical and cubic nanoparticles.

Mesh parameter	Input value
maximum element size	$\frac{e_{\mathrm{c}}}{240}$
minimum element size	$\frac{e_{\mathrm{c}}}{20000}$
maximum element growth rate	1.1
curvature factor	0.2
resolution of narrow regions	1

The meaning of curvature factor, minimum element size and resolution of narrow regions are not significantly important here, as the mesh is mainly determined by the number of elements defined at the edges, maximum element size and maximum element growth rate.

Employing these mesh settings, simulations for different electrode sizes were performed. The dimensionless steady-state flux for a cube (J_{c}) with

$$J_{\mathrm{c}} = \frac{j}{e_{\mathrm{c}} c D}$$

where the edge length e_c was identified for different parameters, is presented in Table S3.

Table S3. Dimensionless diffusional steady-state fluxes for various cubic nanoparticles.

e_{c} (nm)	J_{c}
2	5.4045
26.5	5.4051
51	5.4081
75.5	5.4029
100	5.4038

Although the model is newly meshed for every size, the dimensionless flux does not vary significantly and allows to estimate the steady state current relation for a cubic particle as

$$I_{\mathrm{ss}} = 5.4\, e_{\mathrm{c}} c D z F$$

where F is Faraday constant and z is the number of transferred electrons. Since the presence of singularities at the edges of the cube cannot be avoided and the hemispheric model does not possess such, an estimation of the error for this dimensionless factor is not trivial. Although the simulation at the hemisphere had an error of less than 0.01 %, this is most likely not the case for the cubic nanoparticle. Nevertheless, it is very unlikely that the error significantly alters this equation in an experimentally relevant magnitude, therefore it was used for an analysis of experimental impact currents.

For the analysis of direct support effects, the same simulation geometry was employed, although with a different electrode surface. Here, only the sides of the cube up to a certain height were treated as an electrode with a concentration of zero. Consequently, the height of the active area around the cube was altered, while maintaining the mesh density from the former cube edge at the newly emerging boundary of active and inactive area. The results are shown in Figure 5 in the main text.

SI-II) Conversion of nano-impact data to size distribution

$$I_{ss} = 5.4\ e_c z F D_{O_2} c_{O_2}$$

z: 4
F: 96485 C mol^{-1}
D_{O_2}[1]: 1.90×10^{-9} m^2 s^{-1}
c_{O_2}[1]: 1.21 mol m^{-3}

During OER nano-impact experiments, also the target microelectrode can produce O_2. The concentration of the latter can be estimated by the background current. Considering, as an example, that in Figure S7 a 25 μm diameter Pt microelectrode shows at 0.95 V a *ca.* 1200 pA background current, then:

$$c_{O_2}^{bg} = \frac{I}{4zFD_{O_2}r} = \frac{1200\ \text{pA}}{4 \times 4 \times 96485\ \text{C mol}^{-1} \times 1.90 \times 10^{-9}\ \text{m}^2\,\text{s}^{-1} \times 12.5\ \mu\text{m}} = 0.033\ \text{mol m}^{-3}$$

In our previous work [2], a 0.068 mol m^{-3} background concentration of oxygen was proven to be negligible in the estimation of nanoparticle dimension from impact current data. Considering this, we do not consider the background oxygen concentration stemming from the supporting electrode and take 1.21 mol m^{-3} as the maximum oxygen concentration c_{O_2} to calculate the size of cubes by means of:

$$e_c = \frac{I}{5.4\ zFD_{O_2}c_{O_2}} = \frac{I}{5.4 \times 4 \times 96485\ \text{C mol}^{-1} \times 1.90 \times 10^{-9}\ \text{m}^2\,\text{s}^{-1} \times 1.21\ \text{mol m}^{-3}} = \frac{I(\text{pA})}{4.8}$$

A similar reasoning might be tried also at 1.0 V, but in that case the background current on a Pt microelectrode is much higher (ca. 3000 pA and 0.083 mol m^{-3}) and might not be negligible anymore. In the case of higher background O_2 production a decrease in the step height of the impact currents is expected [2]. Such trend is indeed in line with the decrease in the average step current observed in Figure 4a. However, actual numbers are difficult to compare as continuous O_2 bubble formation (suggested from the increasing background noise level with potential on Pt microelectrode, see Figure 3a and S7) might compensate such effect by helping to remove excess oxygen from the surrounding of the supporting electrode. Therefore, we attribute to the 0.95 V investigation the more reliable evaluation of the nanoparticle dimension distribution.

Comparison to TEM sizing

The size derived from nano-impact results is larger than that from TEM results. This may originate from the dynamic character of the nano-impact system in contrast to TEM analysis. For example, during nano-impacts contact between the electrode and the cube might be made across a cube edge or a corner, resulting in the activity of 6 catalytically active cube faces instead of the previously assumed 5 accessible cube faces that were seen in all TEM images of the surface immobilized cubes. If 6 cube faces are catalytically accessible during a nano-impact, then the detected average current would be increased by roughly 15%, and the above-described estimation of the cube edge length would overestimate the impact-based edge length accordingly.

Two additional effects might contribute to the observed difference between the dynamic nano-impact and the static TEM data. Firstly, the steady state current at the cubes appears to be related to the solubility limit of oxygen in aqueous solutions [2]. This limit corresponds to an equilibrium value which might, however, be locally overcome by supersaturation. In this case, the conversion rate per cube would be limited by a larger maximum O_2 concentration, allowing the observation of larger current signals. Secondly, only current steps clearly exceeding noise level were included in the histograms. Therefore, the increasing background noise level with increasing applied potentials can effectively act as a “filter” against low-current events [3]. Both these effects can shift the measured average current to slightly higher values and cause an overestimation of the nano-impact based calculation of the cube edge length. While all three aspects may limit the precession of nano-impact based sizing of nanocubes, the support effect on nano-electrocatalysis discussed in this work can be unambiguously accessed.

SI-III) Calculation of turnover frequencies (TOFs)

SI-III.1. TOFs determined from ensemble studies

Three methods were used to calculate the TOF values, either, considering all the Co atoms, only the Co atoms at surface of the nanocubes, or just the number of Co atoms contributing to the anodic CV peak at ca. 0.52 V *vs.* Ag/AgCl (which we attribute to $Co^{3+/4+}$ oxidation). The calculations are showcased below in the sections 1.1 to 1.3, while the actual data are reported in section 1.4 Table S4.

SI-III.1.1 All Co atoms are accessible for OER

A single unit cell of spinel Co_3O_4 contains 18 Co atoms and has a volume 0.13 nm^3 and mass 8.0×10^{-22} g according to the simulated structure [4].

To obtain a mass loading of 280 µg cm^{-2}, 0.02 mg Co_3O_4 cubes were drop-cast on a rotating disk electrode.

The corresponding number of moles of Co atoms is: $\mathrm{n(Co)} = \frac{18}{N_A} \times \frac{\mathrm{m}(Co_3O_4)}{8.0\times10^{-22}} = 7.5 \times 10^{-7}\ mol$

$\mathrm{TOF} = \frac{\mathrm{I}}{n(Co)\times4F} = 3.5 \times \mathrm{I}\ (A)\ \ \mathrm{s^{-1}}$

(I: Current at a certain potential, A; F: 96485 C mol^{-1})

For a loading of 2.8 µg cm^{-2}, $\mathrm{TOF} = \frac{\mathrm{I}}{n(Co)\times4F} = 3.5 \times 10^2 \times \mathrm{I}\ (A)\ \ \mathrm{s^{-1}}$

SI-III.1.2 Surface Co atoms are accessible for OER

For cubic particles, we hypothesize (100) to be the mainly exposed plane on the surface.

Co atoms on (100) plane: $N_{100} = 4\times1/4 + 1\times1 = 2$ [5]

Area of (100) plane: $A_{100} = a^2 = 0.65\ nm^2$

(a = b = c= 0.8084 nm, JCPDS no. 42-1467, space group Fd3m)

Surface Co atoms on a cube (5 faces-exposed to electrolyte):

$n_{Co} = A_{cube} \times N_{100} / (A_{100} \times N_A) = 2\times5\times(8.7\ nm)^2 / (0.65\times6.0\times10^{23}) = 1.9\times10^{-21}$ mol

Therefore, in the case of 280 µg cm^{-2} load (0.02 mg Co_3O_4):

The number of cubes are: $N = \frac{0.02\ mg}{m_{cube}} = \frac{0.02\ mg}{6.1\ g\ cm^{-3} \times (8.7\ nm)^3} = 5.0\times10^{12}$

Surface Co atoms: $n(Co) = N \times n_{Co} = 5.0\times10^{12} \times 1.9\times10^{-21}$ mol $= 9.5\times10^{-9}$ mol

$$\mathrm{TOF} = \frac{\mathrm{I}}{n(Co) \times 4F} = 2.7 \times 10^2 \times \mathrm{I}(A)\ s^{-1}$$

For a loading of 2.8 µg cm^{-2}, $\mathrm{TOF} = \frac{\mathrm{I}}{n(Co)\times4F} = 2.7 \times 10^4 \times \mathrm{I}(A)\ s^{-1}$

In the case of 6 faces-exposed to electrolyte, the TOF calculations are shown as:

For a loading of 280 µg cm^{-2}, $\mathrm{TOF} = \frac{\mathrm{I}}{n(Co)\times4F} = 2.2 \times 10^2 \times \mathrm{I}(A)\ s^{-1}$

For a loading of 2.8 µg cm^{-2}, $\mathrm{TOF} = \frac{\mathrm{I}}{n(Co)\times4F} = 2.2 \times 10^4 \times \mathrm{I}(A)\ s^{-1}$

SI-III.1.3 Co atoms contributing to the oxidation peak are accessible for OER

Integrating the anodic peaks assigned to $Co^{3+/4+}$ oxidation in Figure S3, represents another common treatment to estimate the number of OER-active Co sites.

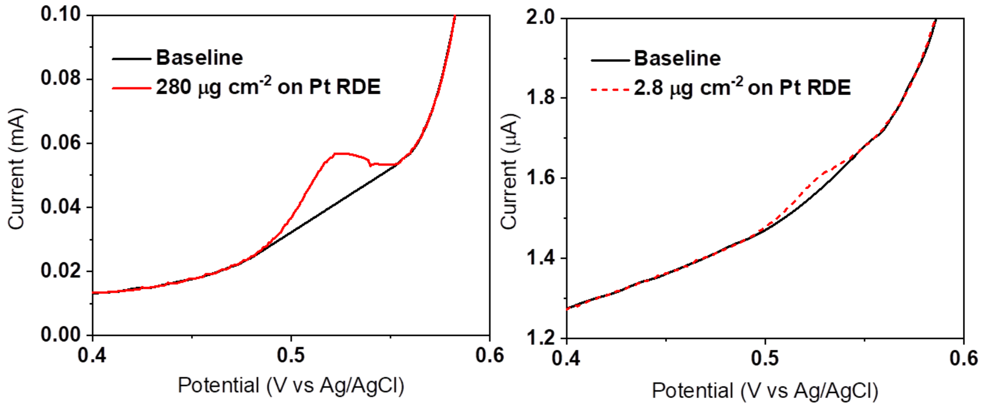

Figure S3. Anodic peaks assigned to $Co^{3+/4+}$ oxidation in Co_3O_4 cubes at loadings of 280 and 2.8 μg cm^{-2} on a Pt RDE.

Taking 280 μg cm^{-2} as an example:

$A_{peak} = 5.2\times10^{-4}$ mA V

$Q_{peak} = A_{peak}$/scan rate = 5.2×10^{-4} mA V / 5 mV s^{-1}= 1.0×10^{-4} C

$n_{Co} = Q_{peak}/(q_e\times N_A) = 1.1\times10^{-9}$ mol (q_e is 1.6×10^{-19} C)

Table S4. Summarized results for the amount of Co atoms contributing to the oxidation peak.

	Area (mA V)	Charge Q (C)	n_{Co} (mol)
280 μg cm^{-2}	5.2×10^{-4}	1.0×10^{-4}	1.1×10^{-9}
2.8 μg cm^{-2}	9.8×10^{-7}	2.0×10^{-7}	2.0×10^{-12}

For a loading of 280 μg cm^{-2}, $\mathrm{TOF} = \frac{\mathrm{I}}{n(Co)\times 4F} = 2.4\times10^{3}\times \mathrm{I}(A)\ s^{-1}$

For a loading of 2.8 μg cm^{-2}, $\mathrm{TOF} = \frac{\mathrm{I}}{n(Co)\times 4F} = 1.3\times10^{6}\times \mathrm{I}(A)\ s^{-1}$

SI-III.1.4 Summarized TOF values estimated from ensemble studies with a Pt RDE

Table S5. Summarized TOF values from ensemble studies with a Pt RDE.

Ensemble studies			TOF of ensemble studies (s^{-1})							
E (V vs Ag/AgCl)	I* (mA)		Case 1 #		Case 2 #				Case 3 #	
	280 μg cm^{-2}	2.8 μg cm^{-2}	280 μg cm^{-2}	2.8 μg cm^{-2}	(6 faces) 280 μg cm^{-2}	(6 faces) 2.8 μg cm^{-2}	(5 faces) 280 μg cm^{-2}	(5 faces) 2.8 μg cm^{-2}	280 μg cm^{-2}	2.8 μg cm^{-2}
0.75	5.8	0.2	2.0×10^{-2}	5.6×10^{-2}	1.3	3.6	1.6	4.3	1.4×10^{1}	2.1×10^{2}
0.8	/	0.5	/	0.17	/	1.1×10^{1}	/	1.3×10^{1}	/	6.2×10^{2}
0.85	/	1.0	/	0.33	/	2.2×10^{1}	/	2.6×10^{1}	/	1.2×10^{3}
0.9	/	1.5	/	0.52	/	3.4×10^{1}	/	4.1×10^{1}	/	2.0×10^{3}
0.95	/	2.1	/	0.72	/	4.7×10^{1}	/	5.6×10^{1}	/	2.7×10^{3}

*Current values of ensemble studies are for 95% iR-corrected data. For 280 μg cm^{-2}, when potentials are higher than 0.75 V *vs.* Ag/AgCl, the obtained current may be disturbed by evolving gas bubbles (see Figure 2a).

Case 1: All Co atoms are active

Case 2: Surface Co atoms are active (6 and 5 faces exposed to electrolyte)

Case 3: Co atoms contributing to the Co^{3+}/Co^{4+} oxidation peak are active

SI-III.2. TOFs determined from nano-impact studies

For cubic particles, we hypothesize (100) to be the mainly exposed plane on the surface, in line with TEM characterization (see Figure 1, main text).

Co atoms on (100) plane: $N_{100} = 4\times1/4 + 1\times1 = 2$ [5]

Area of (100) plane: $A_{100} = a^2 = 0.65\ nm^2$

(a = b = c = 0.8084 nm, JCPDS no. 42-1467, space group Fd3m)

$n_{Co} = A_{cube} \times N_{100} / (A_{100} \times N_A) = 2\times6\times(8.7\ nm)^2/ (0.65\times6.0\times10^{23}) = 2.3\times10^{-21}$ mol

$$\mathrm{TOF} = \frac{\mathrm{I}}{n(Co) \times 4F} = 1.1 \times 10^3 \times \mathrm{I}(pA)\ s^{-1}$$

To be consistent with the diffusion simulation to individual nanocubes, 5 electrolyte-exposed faces of a cube were also taken into consideration.

$n_{Co} = A_{cube} \times N_{100} / (A_{100} \times N_A)=2\times5\times(8.7\ nm)^2/ (0.65\times6.0\times10^{23}) = 1.9\times10^{-21}$ mol

$$\mathrm{TOF} = \frac{\mathrm{I}}{n(Co) \times 4F} = 1.3 \times 10^3 \times \mathrm{I}(pA)\ s^{-1}$$

The corresponding results are summarized in Table S-V.

Table S6. Summarized TOF values estimated from collisions of single cubes on a potentiostated Pt microelectrode.

Nano-impact		TOF of single cubes (**10^4** s^{-1})	
E (V vs Ag/AgCl)	I (pA)	6-faces	5-faces
0.8	10.4	1.2	1.4
0.85	12.7	1.4	1.7
0.9	34.6	3.9	4.6
0.95	54.3	6.1	7.3

SI-IV) Additional supplementary figures

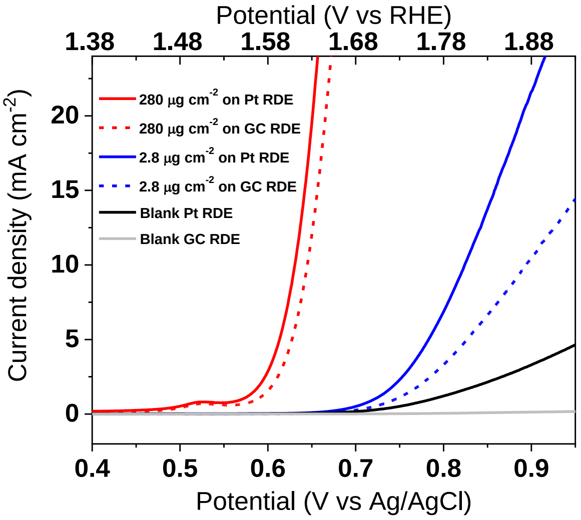

Figure S4. RDE characterization of Co_3O_4 nanocube ensembles employing different support materials at 1600 rpm; LSV curves of 2.8 µg cm^{-2} and 280 µg cm^{-2} Co_3O_4-catalyst films drop-cast on Pt and GC RDEs without further additives at 5 mV s^{-1} (95% iR compensation).

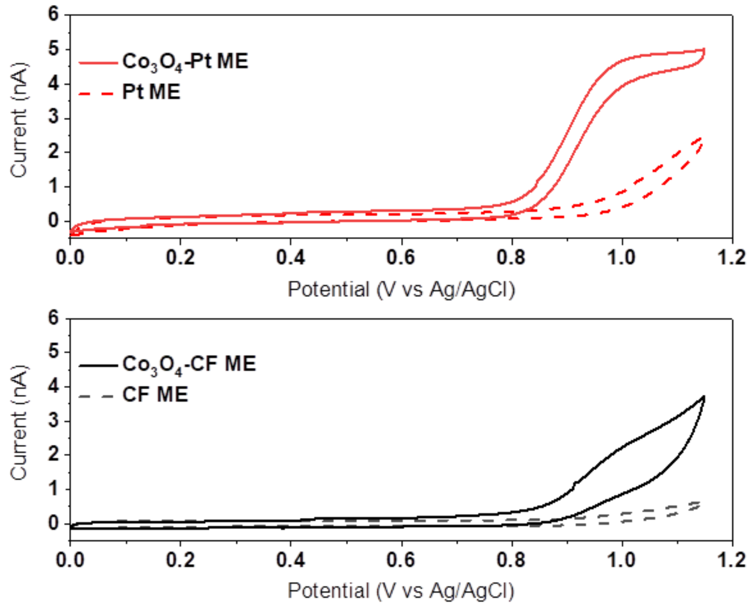

Figure S5. CV curves of Pt and carbon fiber (CF) microelectrodes (ME) before and after loading of Co_3O_4 nanocubes by dipping into a nanocube suspension, measured in an aqueous 0.6 mM KOH and 0.1 M KCl solution.

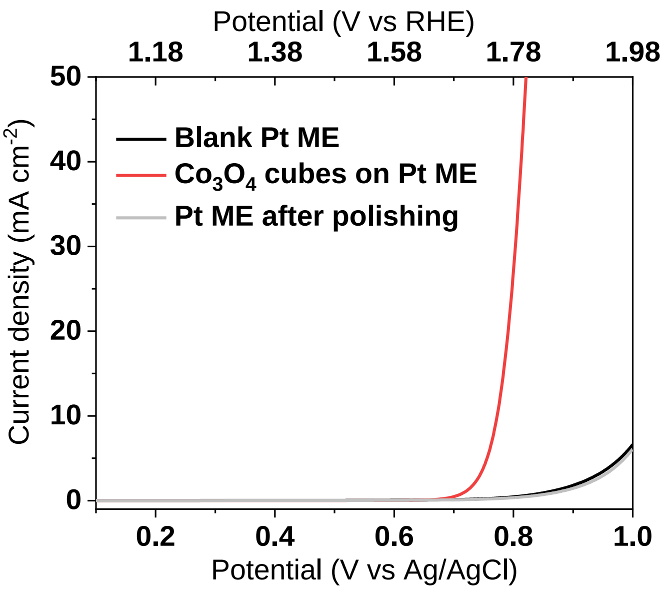

Figure S6. Pre-characterization of a self-made Pt microelectrode to test the electrode suitability for nano-impact experiments. LSV curves of a Pt microelectrode in 0.1 M KOH solution after cleaning, Co_3O_4 nanocubes loading, and repeated polishing/cleaning to remove the nanocubes.

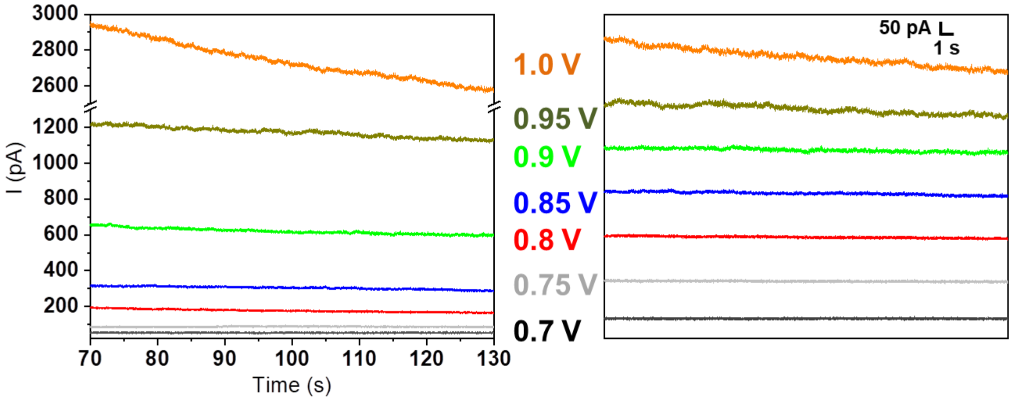

Figure S7. Chronoamperometric blank tests of a Pt microelectrode in 0.1 M KOH solution at different potentials *vs*. Ag/AgCl. On the right, we report the data at the same scale as the data presented in Figure 4c.

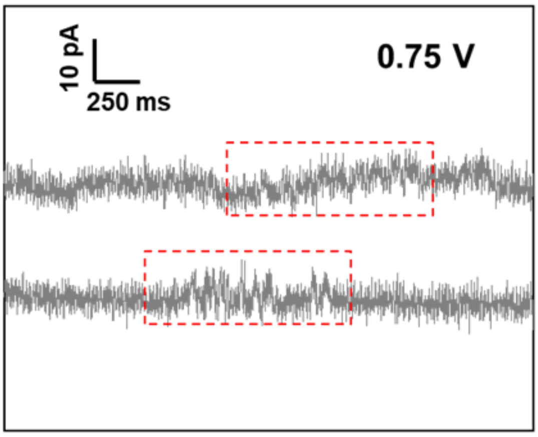

Figure S8. Representative chronoamperograms of Co_3O_4 nanocube-impact measurements with a Pt microelectrode potentiostated at 0.75 V. Red rectangles suggest areas where nano-impact-related current signals may be visible. However, as such signals are not clearly distinguishable from the background noise, they are not considered in the histograms presented in Figure 4.

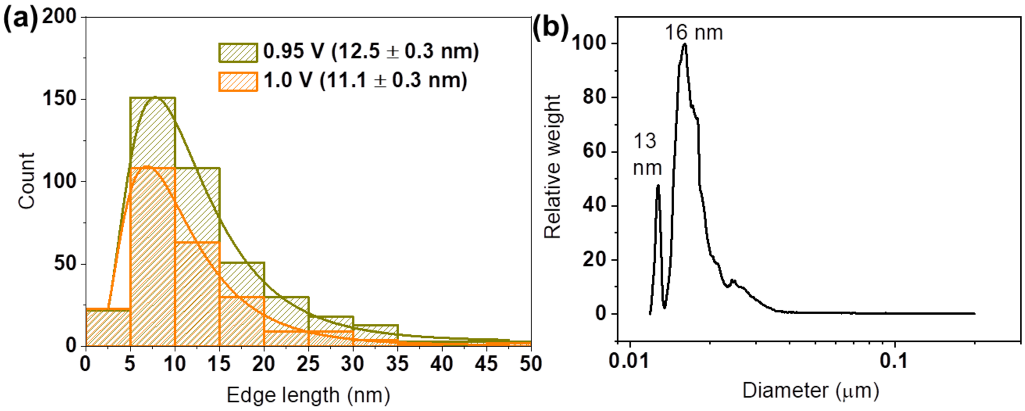

Figure S9. Size distributions of Co_3O_4 cubes derived from (a) nano-impact measurements at 0.95 V and 1.0 V *vs.* Ag/AgCl and (b) disc centrifuge measurements.

As discussed in SI-II, the apparent decrease of nanoparticle mean size at higher potential (1.0 V) may be caused by the increased background noise ruling out the detection of current signals originating from smaller nanocubes hitting the Pt target microelectrode.

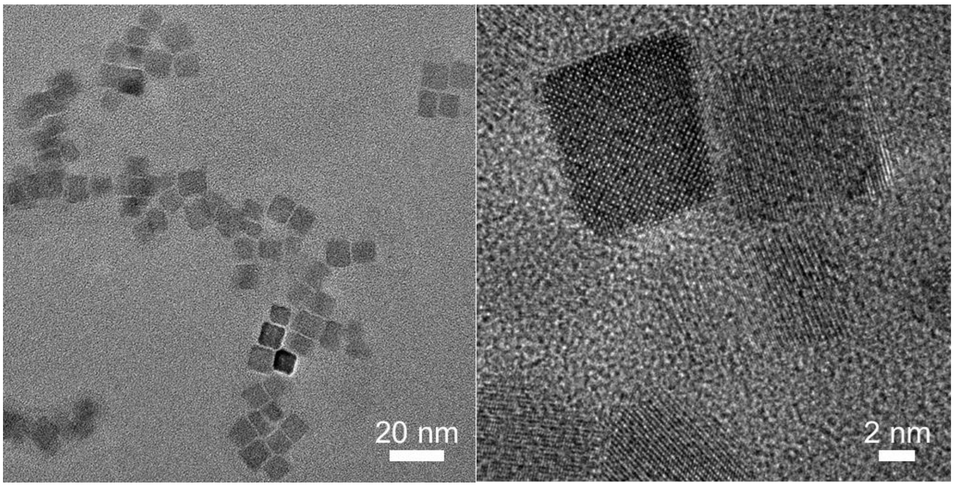

Figure S10. TEM images of Co_3O_4 nanocubes after OER-electrocatalysis. As prepared cubes were sparsely drop-cast on a Au TEM grid. The catalyst modified grid was then immersed into 0.1 M KOH and subjected to OER for 15 minutes at a current density of 10 mA cm^{-2} (normalized to the geometric area of the Au grid). This Au grid was then imaged by bight field TEM, as described above, showing that the size and cubic shape of the particles were maintained after OER catalysis.

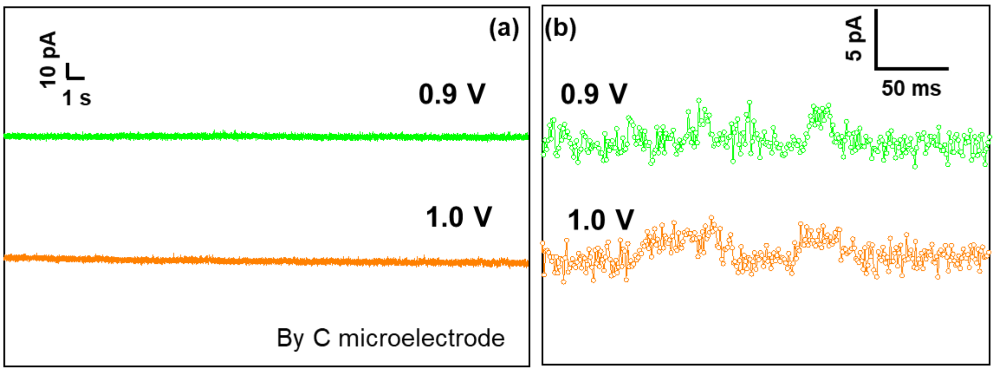

Figure S11. (a) Representative chronoamperograms (offset vertically for better comparison), (b) enlarged impact signals of Co_3O_4 nanocubes impacting a carbon microelectrode at 0.9 and 1.0 V.

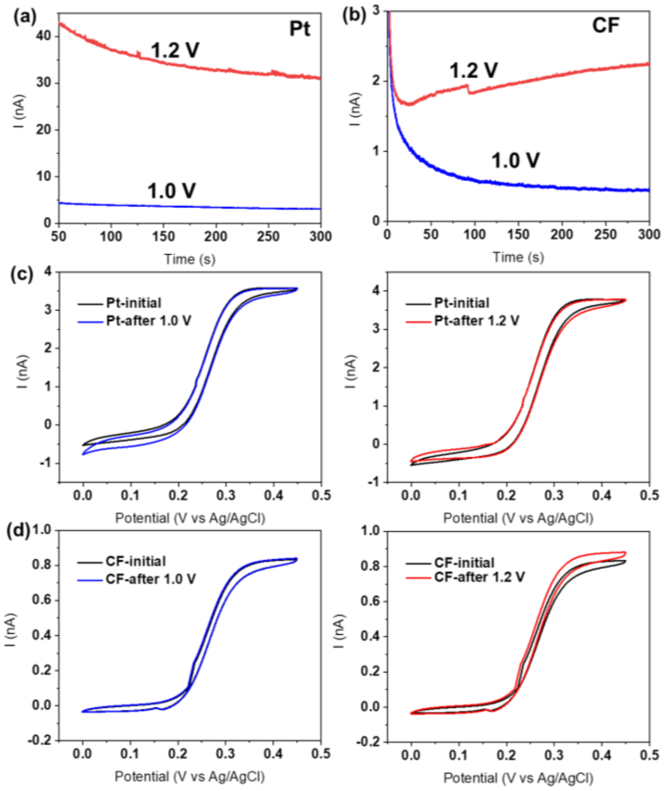

Figure S12. Chronoamperometry of (a) Pt and (b) carbon fiber (CF) microelectrodes in 0.1 M KOH solution at 1.0 and 1.2 V *vs.* Ag/AgCl for 5 min and CV curves in aqueous 1 mM ferrocenedimethanol and 0.1 M KCl solution of (c) Pt and (d) carbon fiber microelectrodes before and after the chronoamperometric measurement.

To test the stability of Pt and carbon fiber microelectrodes, both chronoamperometry and CV experiments have been performed. During chronoamperometry with a carbon fiber microelectrode in 0.1 M KOH at 1.2 V *vs.* Ag/AgCl, the current increases significantly (see Figure S12b), suggesting degrading electrochemical processes (*i.e.* carbon corrosion). In addition, CV curves collected in 1 mM ferrocenedimethanol solutions before and after chronoamperometry at 1.2 V hint towards surface modification of the carbon fiber microelectrode as steady state currents vary (Figure S12d). In contrast, measurements utilizing carbon fiber electrodes at 1.0 V *vs.* Ag/AgCl or Pt microelectrode at 1.0 V and 1.2 V do not indicate surface degradation. Note that while this strongly suggests that degradation of the target electrode is unlikely causing the observed support-effect in our nano-impact experiments, it is difficult to fully exclude changes of the target electrode material.

References

1. Davis, R.E.; Horvath, G.L.; Tobias, C.W. The Solubility and Diffusion Coefficient of Oxygen in Potassium Hydroxide Solutions. *Electrochim. Acta* **1967**, *12*, 287-297.
2. El Arrassi, A.; Liu, Z.; Evers, M.V.; Blanc, N., Bendt, G.; Saddeler, S.; Tetzlaff, D.; Pohl, D.; Damm, C.; Schulz, S; et al. Intrinsic Activity of Oxygen Evolution Catalysts Probed at Single $CoFe_2O_4$ Nanoparticles. *J. Am. Chem. Soc.* **2019**, *141*, 9197-9201.
3. Lees, J.C.; Ellison, J.; Batchelor-McAuley, C.; Tschulik, K.; Damm, C.; Omanović, D; Compton, R.G. Nanoparticle Impacts Show High-Ionic-Strength Citrate Avoids Aggregation of Silver Nanoparticles. *ChemPhysChem* **2013**, *14*, 3895-3897.
4. Jain, A., Ong, S.P.; Hautier, G.; Chen, W.; Richards, W.D.; Dacek, S.; Cholia, S.; Gunter, D.; Skinner, D.; Ceder, G.; et al. The Materials Project: A Materials Genome Approach to Accelerating Materials Innovation. *APL Mater*. **2013**, *1*, 011002.
5. Gao, R.; Zhu, J.; Xiao, X.; Hu, Z.; Liu, J.; Liu, X. Facet-Dependent Electrocatalytic Performance of Co_3O_4 for Rechargeable Li-O_2 Battery. *J. Phys. Chem. C* **2015**, *119*, 4516-4523.
